# Retraction: Transversus Abdominis Plane Block Versus Local Anesthetic Wound Infiltration for Postoperative Analgesia in Adult Patients Undergoing Hernia Repair in Daycare Procedure: A Randomized Control Trial

**DOI:** 10.7759/cureus.r45

**Published:** 2022-03-17

**Authors:** Sidra Mukhtar, Madeha Ishag Adam, Estefania Martinez-Jimenez, Hamza Naseem, Insiya Sherawala, Shivani M Mehta, Fares Mohammed Saeed Muthanna, Abdul Muqtadir, Rahil Barkat

**Affiliations:** 1 Internal Medicine, Mayo Hospital, Karachi, PAK; 2 Neurogenerative Disease, Fujian Medical University, Fuzhou, CHN; 3 Medical School, Universidad Autonoma del Estado de Hidalgo, Pachuca, MEX; 4 Medical School, Jinnah Medical and Dental College, Karachi, PAK; 5 Medical School, Bharati Vidyapeeth Deemed Medical College, Pune, IND; 6 Medical School, Xavier University School of Medicine, Oranjestad, ABW; 7 Department of Pharmacy, Walailak University, Nakhon Si Thammarat, THA; 8 Medicine and Surgery, Shadan Institute of Medical Sciences, Dr. NTR University of Health Sciences, Hyderabad, IND; 9 Indus Hospital Research Center, The Indus Hospital, Karachi, PAK

This article has been retracted due to the unknown origin of the data, lack of verified IRB approval, and purchased authorships. The primary author, Rahil Barkat was involved in data theft and misuse in two recently published Cureus articles, which have since been retracted.

As the origin of this article’s data and verified IRB approval cannot be confirmed, we have made the decision to retract this article. Cureus has confirmed that the co-authors were asked by Mr. Barkat to proofread the article and provide payment in exchange for authorship. (Proofreading is an insufficient contribution to warrant authorship as defined by ICMJE.) These payments were made in the guise of “editing fees” but greatly exceed any editing fees paid to Cureus. While these authors may have been defrauded by Mr. Barkat, they remain complicit due to their lack of honest contributions to the article.

